# Detection of Pediatric Dental Caries in Panoramic Radiograph Using Deep Learning: A Benchmark Study on MD-OPG

**DOI:** 10.3390/s26082481

**Published:** 2026-04-17

**Authors:** Hadi Rahimi, Seyed Mohammadrasoul Naeimi, Shayan Darvish, Bahareh Nazemi Salman, Parvin Razzaghi, Ionut Luchian, Dana Gabriela Budala

**Affiliations:** 1 Department of Computer Science and Information Technology, Institute for Advanced Studies in Basic Sciences (IASBS), Zanjan 45137-66731, Iran; hadirahimi@iasbs.ac.ir (H.R.); p.razzaghi@iasbs.ac.ir (P.R.); 2 School of Dentistry, Zanjan University of Medical Sciences, Zanjan 4513956184, Iran; emadnaeiminasir@gmail.com; 3 Private Practice, Tucson, AZ 85741, USA; sdarvish@umich.edu; 4 Department of Pediatrics, School of Dentistry, Zanjan University of Medical Sciences, Zanjan 4513956184, Iran; 5 Grigore T. Popa University of Medicine and Pharmacy, 700115 Iasi, Romania; ionut.luchian@umfiasi.ro (I.L.); dana-gabriela.bosinceanu@umfiasi.ro (D.G.B.)

**Keywords:** artificial intelligence, panoramic radiograph, pediatric dental caries, deep learning, medical imaging

## Abstract

Early detection of dental caries in children is critical to prevent irreversible tooth damage and guarantee optimal oral health outcomes. However, interpreting pediatric panoramic radiographs throughout the mixed dentition stage remains a very challenging task due to overlap in anatomical structures and developmental variability. This complexity underscores the need for well curated, representative datasets that enable the development of reliable computer-aided diagnostic models. Herein, this study introduces the Mixed Dentition Orthopantomogram Dataset, a newly developed, publicly available dataset of children that was carefully labeled by dental specialists to identify proximal and occlusal caries regions in the range of 3–12 years. To evaluate the dataset’s applicability for artificial intelligence research, we benchmarked it using both classification and segmentation models. A patch-based classifier achieved an average AUC of 0.89 and Recall 0.85 in distinguishing healthy and carious regions. For segmentation, we evaluated U-Net and Attention U-Net with multiple loss functions, and the Attention U-Net trained with Focal loss achieved the best Dice score of 0.94. Collectively, these findings support the dataset’s utility for pediatric caries analysis and demonstrate the viability of deep learning approaches for mixed dentition panoramic imaging.

## 1. Introduction

Dental caries remains one of the most prevalent chronic diseases worldwide, affecting individuals of all ages and leading to significant public health challenges [1]. Early detection is essential to prevent irreversible damage, especially in growing patients. Radiographic imaging plays a central role in caries diagnosis and treatment planning. In particular, imaging aids in detecting lesions not visible during clinical examination, making it indispensable for screening and follow-up [2]. Up to 12 years old, children enter a transitional stage called mixed dentition, in which primary and permanent teeth coexist [3]. This period presents unique diagnostic difficulties due to rapid developmental changes, overlapping anatomical structures, and variable eruption patterns. The complexity of interpreting radiographs during this stage often leads to diagnostic uncertainty, especially for less experienced dentists [4]. While periapical (PA) and bitewing radiographs offer higher resolution for detecting early-stage lesions, they require patient cooperation and precise positioning, which are often unmet challenges in pediatric populations [5]. In contrast, panoramic orthopantomograms (OPGs) offer a broad overview of the entire dentition in a single image with minimal patient compliance. OPGs are therefore widely used in pediatric dental assessments, especially in less cooperative patients or in routine screening contexts [6,7]. Traditional methods of caries detection, such as visual inspection and manual interpretation of radiographs, are often subjective and prone to error. AI-powered tools have shown promise in addressing these challenges [8]. Studies have demonstrated that AI models, particularly convolutional neural networks (CNNs), can extract complex features and achieve high sensitivity, specificity, and accuracy in detecting caries across different imaging modalities, including OPGs [9]. Despite its potential, the application of AI to pediatric OPGs, particularly during the mixed dentition phase, remains significantly underexplored. One major limitation is the lack of publicly available, well-annotated datasets that represent this complex developmental stage, which restricts model development and benchmarking. Without such resources, AI models cannot be trained or validated effectively for pediatric-specific diagnostic tasks. From a clinical perspective, AI-assisted analysis of mixed dentition OPGs could significantly improve diagnostic support. Automated identification of carious lesions may reduce observer variability, enhance early detection in screening programs, and support dental education. To address this need, we introduce MD-OPG, a publicly available dataset focused on pediatric mixed dentition. This study evaluates the feasibility of automated caries detection on OPGs using MD-OPG and benchmark deep learning models. Our findings provide baseline insights for future research and highlight the potential clinical utility of AI-assisted tools in improving diagnostic consistency, particularly for less experienced clinicians and routine pediatric screenings.

## 2. Materials and Methods

**Figure 1:**
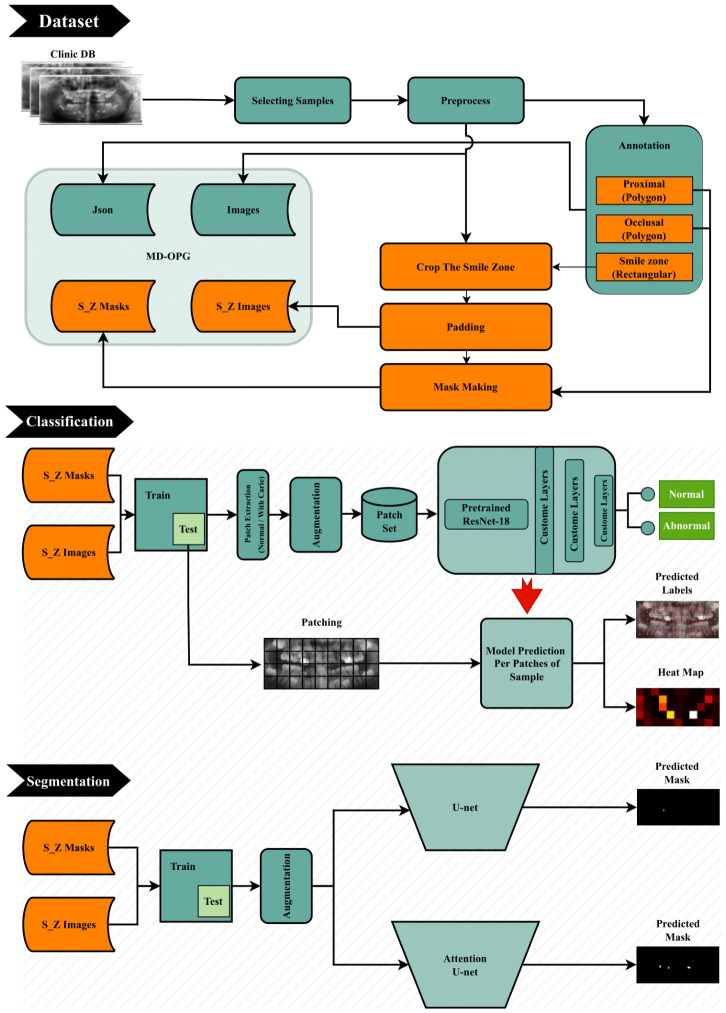
Complete Architecture of the Presented Method.

This study retrospectively used de-identified pediatric OPGs. Also, samples were randomly selected from archival records to create a new AI caries detection dataset. As visible in Figure 1, the first part of this work deals with dataset collection and preparation. In the next phase, this research explores the efficiency of machine learning methods on the proposed dataset by using well-known deep learning architectures within a new analysis pipeline specifically designed for pediatric mixed-dentition OPG images. Although the core models (ResNet-18, U-Net, Attention U-Net) are standard, their adaptation to this dataset—using a patch-based classification strategy and lesion-focused segmentation—represents a methodological contribution in how OPG images of children are processed and evaluated. This stage highlights the dataset’s potential and provides a solid baseline for future work. To this end, we have applied a leading architecture for classification named ResNet [10] with an interesting approach for the approximate determination and display of caries-containing areas. In addition, we have also utilized two widely recognized deep learning models for segmentation: U-Net [11] and Attention U-Net [12]. The first is known for its encoder–decoder architecture with skip connections and is a popular choice for medical image segmentation due to its ability to capture fine-grained details. Attention U-Net builds upon this foundation by incorporating attention mechanisms, allowing the model to focus on the most relevant features in the image. By implementing these models, we aimed to showcase the potential of the MD-OPG for applications that require detailed anatomical delineations.

### 2.1. Dataset

**Figure 2:**
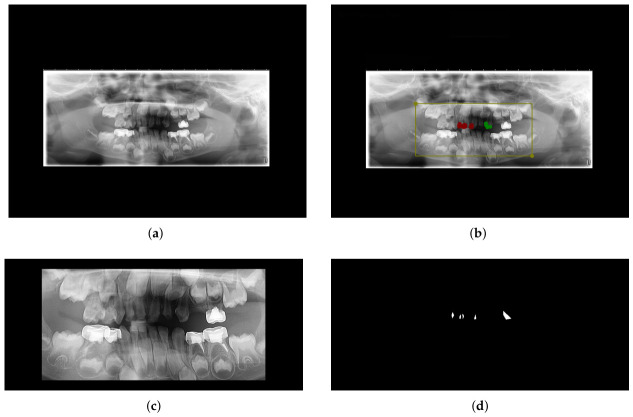
Contents of MD-OPG for each sample or patient. (**a**) Original OPG image. (**b**) An example of an annotation for caries (red and green polygon) and the smile zone (yellow rectangle). (**c**) Smile Zone image (cropped and padded). (**d**) Mask of smile zone image.

Today, dentists use various medical imaging techniques to assess the health of a patient’s teeth before commencing dental procedures. Each image is taken under different conditions: one type is an orthopantomogram (OPG), a panoramic X-ray that provides a wide view of the teeth, upper and lower jaws. This type of imaging does not capture excellent details but can provide useful and complete information, such as the number and position of teeth, hidden teeth, and the progress of growth of teeth and jaws in children [7]. The Mixed Dentition OPG Dataset for AI Research in Pediatric Dental Cariology (MD-OPG) was developed through the collaborative efforts of the Institute for Advanced Studies in Basic Sciences (IASBS) and Zanjan University of Medical Sciences (ZUMS). The dataset was assembled with particular attention to ensuring patient confidentiality and ethical data management under applicable privacy regulations. It is intended to support research on artificial intelligence and computer vision applications within the domain of pediatric dental health, and it forms the basis for the present study. According to Table 1, the dataset specifically targets pediatric patients in the mixed dentition phase, characterized by the simultaneous presence of primary and permanent teeth. As such, the selected panoramic radiograph samples were obtained from individuals between the ages of 3 and 12. Inclusion criteria consisted of digital OPGs with mixed dentition and sufficient image quality for caries annotation, while images with poor quality, absence of target caries, or incomplete dentition were excluded. The data were collected from a single private dental clinic in Zanjan, Iran, and all image selection and annotation were conducted under the supervision of experienced dental specialists. All images were initially annotated by two mid-level annotators trained in dental radiographic interpretation. The annotations were subsequently reviewed and validated by two experienced dental specialists. Any discrepancies were resolved during the expert review process to produce the final consensus annotations used in this study. The resulting dataset consists of 170 samples from both male and female patients. Each sample includes a PNG image accompanied by a JSON file containing annotations of the regions of interest (ROI). The images obtained from the clinic have a fixed size of 1920 × 1535 pixels. However, as shown in Figure 2a, there is a large black border surrounding the main content of the OPG. Excluding this black area, the main OPG content in our images varies in size but maintains a 2:1 aspect ratio. The JSON files include polygons in two classes that precisely indicate the location of caries. These classes are labeled as P, which refers to proximal caries formed between the proximal surfaces of two neighboring teeth, and O, which refers to occlusal caries formed on the occlusal surfaces of the teeth. Carious lesions appear as low-density areas on the tooth structure. This area represents the demineralization and dissolving of hard tissue. In addition, a rectangular area called the “smile zone” is also annotated and labeled as Z. The smile zone term refers to the region encompassing the visible crowns of teeth that have erupted within the oral cavity. The rectangular area, which has varying sizes but maintains a 2:1 aspect ratio, provides the exact coordinates of the region containing the crowns of teeth or the smile zone (defined by the upper left coordinates shown by $x_{min}$ and $y_{max}$, and the bottom right coordinates shown by $x_{max}$ and $y_{min}$). This helps in easily isolating the teeth without including unnecessary details such as the jaw bones. In Figure 2b, you can see that both types of caries, proximal (marked red) and occlusal (marked green), are annotated, and the yellow rectangular box defines the smile zone. Also, Figure 2c corresponds to the “smile zone image” of the original image, which has been cropped and padded. We padded all smile zone samples with particular values (because of their different sizes) to achieve a uniform shape across all samples. Figure 2d is the corresponding mask for the smile zone image, which is prepared in the dataset for samples.

The presented dataset, with its focus on a specific age group, makes it a valuable resource for both AI and computer science researchers as well as dental specialists and students. It helps to strengthen the collaboration between computer science and dental health by creating a bridge between these fields.

### 2.2. Classification

In machine learning for visual data, especially medical images with tiny details, patch-based models are widely used [13]. These models focus on small image regions (patches) to capture local patterns and textures. This helps in learning important features and improves performance. When the region of interest is known, features can be easily extracted to support more accurate learning. To this end, the proposed method for classifying the patches follows three main steps. First, we extract 2 group patches from the input smile zone image from the ROI (abnormal) and normal areas. In the second step, we use augmentation techniques to increase the number of abnormal samples because these patches are significantly lower than normal patches. This is beneficial in two ways: it enhances the training process by providing more abnormal samples for the model and ensures a balance in the patch set in both classes. Finally, we train our model using the provided patch set to classify samples as either containing caries or being healthy. To recap, assume the input patch is $x\in I\;R^{p\times q}$ and the corresponding label of each patch is 0 or 1. The goal is to design a classifier function, shown by *f*, which maps the input patch to the label space $f:I\;R^{p\times q}\;->I\;R^{2}$, including normal (healthy tooth) and abnormal (with caries).

#### 2.2.1. Patch Extraction

Extracting suitable patches for training the model can greatly improve the results, so this step should be done carefully. We know that in OPG images, there are other features besides teeth, such as bone and skull tissue. Given the smile zone coordinates in our dataset, we can simply crop our images to focus the patch extraction area on the teeth region only. However, since each patient has a unique skull shape and dental structure, the cropped smile zone will not be uniform in size and defined as $I_{s}\in I\;R^{h\times w}$, which is h>p & w>q. In the proposed approach, a patch size of 64×64 is used, and a different number of patches is extracted depending on the size of each smile zone. Algorithm 1 provides a pseudo-code representation of the patch extraction process.
**Algorithm 1** Patch Extraction**Require:** Image $I_{s}$, Mask $I_{m}$, Patch Size (p,q=64) **Ensure:** Normal_Patches, Abnormal_Patches
  1:
Initialize Normal_Patches ← []
  2:
Initialize Abnormal_Patches ← []
  3:
**for **i=0 **to** h−p **step** 32 **do**
  4:
      **for** j=0 **to** w−q **step** 32 **do**
  5:             Patch_S $\leftarrow I_{s}\left[i:i+p,j:j+q\right]$
  6:             Patch_M $\leftarrow I_{m}\left[i:i+p,j:j+q\right]$
  7:             **if** any value in Patch_M is non-zero **then**
  8:                   Append Patch_S to Abnormal_Patches
  9:             **else**
10:                   Append Patch_S to Normal_Patches
11:             **end if**
12:       **end for**
13:
**end for**
14:
**return** Normal_Patches, Abnormal_Patches


#### 2.2.2. Network Architecture: Res-Net

Artificial Neural Networks (ANNs) are powerful tools for extracting features from input data. Among these, the Convolutional Neural Network (CNN) is a prominent type that utilizes convolutional kernels to slide over the input matrix and extract comprehensive features. The ResNet convolutional network, a remarkable innovation in deep learning [10], gained significant attention for addressing the vanishing gradient problem by introducing skip connections. The standard blocks in ResNet design consist of various operations such as convolution, batch normalization, and the ReLU activation function. In the classification task, we selected the ResNet-18 architecture for feature extraction from patches, leveraging the knowledge of a pre-trained network on the ImageNet [14]. Additionally, by excluding the top layers that were pre-trained on ImageNet, we added custom layers tailored to our specific task. The custom layers start with a global average pooling layer to reduce spatial size. Then, the data passes through several dense layers with ReLU activation. Dropout is used between layers to prevent overfitting, and the final layer uses softmax to give a probability output for two classes.

### 2.3. Segmentation

Medical images naturally have a more complex structure compared to other types of images, making it very challenging to extract distinct features from surrounding tissues [15]. For this reason, the main goal of the U-Net designers was to create an optimized architecture for feature extraction and segmentation with precise boundaries. This architecture is also recognized as one of the key benchmarks in medical image analysis due to its strong performance in situations with limited datasets. Given the scarcity of labeled data in this field, this is a remarkable feature [16]. Since U-Net performs well in segmenting fine structures in medical images, we use it for MD-OPG, which includes small and subtle dental caries.

#### 2.3.1. Network Architecture: U-Net

As the name suggests, U Net has a U-shaped structure with two main parts: a contracting path (encoder) and an expansive path (decoder). The encoder reduces the spatial resolution while increasing the number of channels, allowing the network to capture high-level contextual features. The decoder then gradually restores the resolution using the features from the encoder’s final layer. Skip connections link corresponding layers from the encoder to the decoder, helping retain spatial information lost during down-sampling. In the original U-Net, the contracting path includes five encoder blocks with the same structure but increasing filter sizes. The expansive path includes four decoder blocks. Each decoder up-samples the features using an up-convolution layer and applies convolutions to reduce feature depth. At each decoding step, features from the matching encoder block are concatenated with the up-sampled output to preserve spatial details.

#### 2.3.2. Network Architecture: Attention U-Net

This model improves semantic segmentation accuracy by using Attention Gates (AGs) in the expansive path. While the contracting path remains similar to U-Net, the expansive path in Attention U-Net differs by passing the features from each up-sampling layer through an Attention Gate (AG) before concatenating them with the encoder features. The AG allows the model to focus on relevant regions by weighting important features higher. The attention gate is mathematically expressed as:

(1) $$g_{i}=\sigma \left(W_{x}x_{i}+W_{g}g+b\right),$$

where $x_{i}$ represents the input feature map from the contracting path, *g* is the gating signal from the corresponding upsampling layer in the expansive path, $W_{x}$ and $W_{g}$ are learnable weight matrices, *b* is a bias term, and σ(·) is the sigmoid activation function, which generates a weight map. This weight map, denoted as $g_{i}$, filters the input feature map by amplifying salient features and suppressing less relevant ones.

After filtering, the output of the attention gate is expressed as:

(2) $$x_{i}^{\prime }=g_{i}⊙x_{i},$$

where ⊙ denotes element-wise multiplication. The filtered features, $x_{i}^{\prime }$, are then concatenated with the upsampled features from the upsampling layer, represented as:

(3) $$z=\mathrm{concat}(x_{i}^{\prime },g).$$

### 2.4. Loss Function

For any machine learning model, good results depend on finding the best parameters and lowering the error. To improve accuracy and reliability, the optimization phase must be carried out efficiently. This includes reducing the loss function, which measures the gap between predictions and true labels, and the loss function guides optimization algorithms like Adam [17] toward better performance. For classification tasks, cross-entropy is a common and effective loss function for both binary and multi-class problems. Suppose *N* represents the number of data samples, $y_{i}\in \left[0,1\right]$ denotes the true label, and $\hat{y}$ represents the predicted label. Under these conditions, the cross-entropy loss function for a single sample is defined as:

$$L_{i}=-\left(y_{i}\log\left({\hat{y}}_{i}\right)+\left(1-y_{i}\right)\log\left(1-{\hat{y}}_{i}\right)\right)$$

The overall loss for the dataset is computed as the average loss across all samples:

(4) $$\begin{array}{c} \mathrm{Cross}\;\mathrm{Entropy}=-\frac{1}{N}\sum_{i=1}^{N}\left(y_{i}\log\left({\hat{y}}_{i}\right)+\left(1-y_{i}\right)\log\left(1-{\hat{y}}_{i}\right)\right) \end{array}$$

In semantic segmentation, choosing the right loss function is the key to improving performance, especially when there is a class imbalance. This imbalance makes the model sensitive to the loss function used. Dice loss [18] is an effective solution designed to handle such cases. It is based on the Dice coefficient and works well with unbalanced data. It evaluates the overlap between the predicted and true regions citedice. Dice loss is defined as:

(5) $$\mathrm{Dice}=1-\frac{2\sum_{i=1}^{n}{\hat{y}}_{i}y_{i}}{\sum_{i=1}^{n}{\hat{y}}_{i}+\sum_{i=1}^{n}y_{i}}$$

Focal loss [19] is a specialized loss function designed to effectively handle class imbalance, particularly in tasks such as object detection and segmentation citefocal. Unlike Dice Loss, which emphasizes improving overlap between predicted and true regions, Focal Loss modifies the standard cross-entropy loss by incorporating a scaling factor to reduce the impact of well-classified examples, thereby focusing more on harder-to-classify samples. In the Focal Loss formula:

(6) $$\mathrm{Focal}=-\sum_{i=1}^{C}\alpha_{i}{\left(1-{\hat{y}}_{i}\right)}^{\gamma }\log\left({\hat{y}}_{i}\right),\;s.t.\;\sum_{i=1}^{C}\alpha_{i}=1.$$

The parameter α acts as a balancing factor to adjust the importance of positive and negative classes, addressing class imbalance at a global level, and *C* refers to the number of classes. The parameter γ, known as the focusing parameter, controls the degree to which the loss is down-weighted for easy-to-classify samples. A higher γ value places more emphasis on hard-to-classify examples, making the loss particularly effective for handling datasets with a significant imbalance between majority and minority classes.

To address the limitations of using Dice Loss or Focal Loss individually, a hybrid approach known as DiceFocal Loss has been introduced. This dual advantage makes it particularly suitable for applications where both imbalanced data distribution and small target regions are significant challenges. The combined loss is mathematically defined as:

(7) $$\begin{array}{c} \mathrm{DiceFocal}=\lambda \cdot \mathrm{Dice}\;\mathrm{Loss}+(1-\lambda )\cdot \mathrm{Focal}\;\mathrm{Loss} \end{array}$$

In (7), λ serves as a weighting parameter, allowing the user to control the relative importance of Dice Loss and Focal Loss based on the specific requirements of the task.

## 3. Results

We implemented a patch-based classification model to evaluate the dataset’s capacity to support image-classification tasks and employed U-Net and Attention U-Net models to assess its suitability for image segmentation tasks. These architectures were selected with two specific objectives. The first goal is to validate the quality and applicability of the proposed dataset by demonstrating its ability to support high-performance models for widely used tasks in medical imaging. The second objective is to provide reproducible benchmark results using state-of-the-art deep learning architectures and offer a foundation for future researchers utilizing the MD-OPG. All analyses and model training were conducted using Python 3.11, with Keras and PyTorch for deep learning, and OpenCV for image preprocessing tasks.

### 3.1. Classification Task: Experimental Setup and Result

As mentioned earlier, the first part of this research aimed to classify the OPG image patches into two groups: those containing caries and those with normal tissue. For this purpose, a ResNet-18 model was trained in 5-fold using patches that were systematically extracted from the smile region. Due to the small and detailed nature of the caries, the patch size was set to 64 × 64 to preserving small lesion details and maintaining sufficient surrounding anatomical context. For the 5-fold cross-validation experiments, the dataset was partitioned at the patient level (i.e., OPG image level) to prevent potential data leakage. Twenty images were set aside as an independent final test set, while the remaining 150 images were used within the cross-validation framework. Because the task was based on patch-level image classification, a distinct patch dataset had to be generated for each fold. To maintain strict separation between training and validation samples, patient IDs corresponding to each fold were predefined prior to patch extraction. Patch extraction was therefore repeated five times, once per fold and ensuring that patches originating from the same patient did not appear in both training and validation sets.

As expected, the training data exhibited notable class imbalance, since only a small fraction of images contained caries while the majority represented the normal class. To mitigate this issue, the majority class was down-sampled and data augmentation was applied to the caries class to improve its representation during training. Following these steps, each fold contained approximately 18,000 patches with an approximately balanced class distribution (around 50% per class). In contrast, no augmentation was applied to the validation data, where the number of caries-containing patches remained roughly one-eighth of the normal class. A comparable imbalance was also present in the independent test set. From the 4030 patches extracted from the test images, only 428 corresponded to the caries class. For model training, a pre-trained ResNet-18 backbone was used in combination with several custom classification layers. The network was trained for 100 epochs with a batch size of 256 using the Adam optimizer and the categorical cross-entropy loss function. The resulting performance metrics are summarized in Table 2.

**Figure 3:**
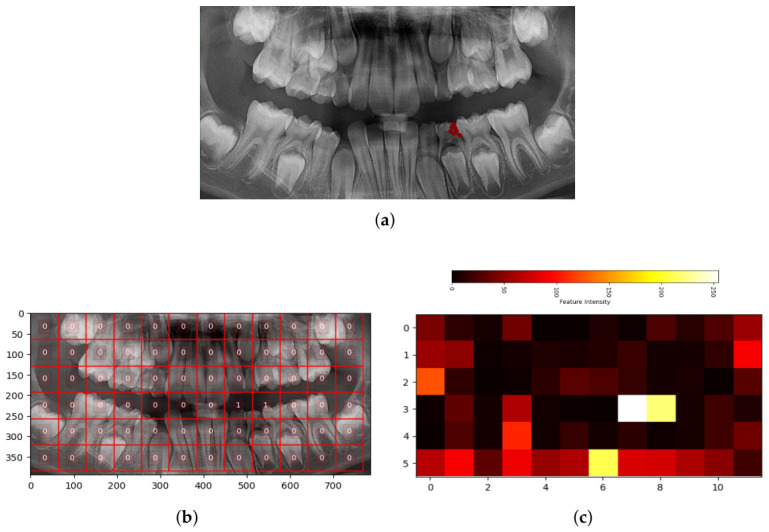
The performance visualization of the patch classifier by generating outputs for all patches of a test smile zone image. (**a**) The OPG image (smile zone), which includes the proximal caries, is annotated with a red polygon. (**b**) Represents the patch classifier’s label predictions. Regions labeled as “1” are identified as patches containing caries. (**c**) Displays a heatmap of features extracted from the layers preceding the classifier. These features are visualized in the range of 0–255.

To visually evaluate the patch classifier, we tested it on a cropped smile zone from the OPG test set that was not used during training. As shown in Figure 3a, this image includes a proximal caries marked with a red polygon. Non-overlapping 64 × 64 patches were extracted from the smile zone and passed through the model, with predictions arranged to match their original positions. In Figure 3b, the model successfully identifies normal and caries areas, labeling them as “0” and “1”, respectively. Figure 3c presents a heatmap of features extracted before the softmax layer, where brighter areas (closer to 255) represent features linked to caries (label 1), helping to visualize how the model interprets these regions.

### 3.2. Segmentation Task: Experimental Setup and Results

Segmentation requires working with full images, which makes unified and careful preprocessing essential to avoid losing critical information. Thankfully, the MD-OPG includes coordinates for the smile zone, allowing the segmentation to focus on relevant areas. The main challenge was that these regions varied in size. Since improper resizing could distort important medical details, we used padding instead. First, we identified the largest smile zone in the dataset and then padded all other images with zeros to match its size (smile zone image). This approach preserved image integrity and standardized all inputs to 440 × 1000 pixels. Corresponding mask images were padded in the same way to ensure alignment with their input images. In training the U-Net network, 40 samples were set aside for testing, and the remaining data was increased to 390 training samples using augmentation. This augmentation included a shift scale, rotation, and horizontal flip. The training was conducted over 100 epochs with a batch size of 8 and a learning rate of 0.0001 using the Adam optimizer. This experiment was performed with three different loss functions (Table 3) to evaluate their impact on the model’s performance. The architecture of the implemented network includes four encoder layers with a kernel size of 2 in the convolutional layers. The number of filters increases from 64 in the first encoder to 512 in the final encoder. Each encoder layer contains ReLU activation, batch normalization, and max pooling operations. On the expansive path, four decoder layers are used to progressively up-sample the spatial dimensions of the features by a factor of 2 using bilinear interpolation. Overall, one encoder and one decoder layer were removed from our architecture compared to the original version To reduce the risk of overfitting on the relatively small dataset, resulting in a lighter model with fewer trainable parameters. This experiment was conducted using three loss functions: Dice Loss, Focal Loss, and DiceFocal Loss. In each epoch, the model’s performance was evaluated using the Dice Score (DS) on the 40 test samples. The Dice coefficient Score measures the overlap between the predicted segmentation and the ground truth, providing a reliable metric for assessing the accuracy and consistency of the model’s predictions across the test set.

Continuing with our research, we experimented using the Attention U-Net architecture. We reserved 40 samples for testing, while the remaining samples were augmented to 390 to enhance the training process. The model was trained for 100 epochs with a batch size of 8 and an Adam optimizer with a 0.0001 learning rate. Up to now, all setups were similar to our U-Net experiment. In our Attention U-Net experiment, no modifications were made to the original architecture: the contracting path consisted of five encoder layers, while the expansive path included four decoder layers plus the final convolution layer to produce a segmentation map. The architecture is composed of convolutional, up-sampling, and attention blocks. Each block is repeatedly positioned with varying inputs and outputs in the architecture flow. The convolutional blocks contain two 2D convolution layers with a kernel size of 3 × 3 and a stride of 1. Each is followed by batch normalization and a ReLU activation function to maintain smooth flow. The up-sampling block begins with an up-convolution layer that increases the spatial dimensions of the feature maps using a scale factor of 2. This is followed by a 2D convolution layer and a ReLU activation function to refine the output. In this implemented architecture, almost all structural components were designed similarly to the original version, except for the number of filters in each block. Due to GPU VRAM limitations, we aimed to reduce the number of parameters. Therefore, we started with 32 filters in the first encoder and increased to 512 filters in the fifth encoder. In experiments using this architecture, we again evaluated the three loss functions (Table 4).

## 4. Discussion

The results of this study demonstrate that the proposed dataset is effective and holds strong potential for use in artificial intelligence research. Its performance, when evaluated using state-of-the-art models, aligns well with key benchmarks commonly used in medical image analysis tasks. During the dataset collection phase, the involvement of dental experts, from sample selection to preprocessing and annotation, made a significant contribution to the improved performance of deep learning-based evaluations. The proposed patch-based detection pipeline demonstrated robust performance in identifying caries regions on panoramic dental OPG images. As summarized in Table 2, the model achieved a mean AUC of 0.879 ± 0.011 across five folds and 0.899 on the independent test set, confirming its strong discriminative capability. The recall (sensitivity) was notably high (0.818 ± 0.024 in cross-validation and 0.857 on the test set), indicating that the system successfully detected the majority of carious patches (Figure 3c) and missed relatively few lesions. This level of sensitivity is particularly meaningful given the challenging nature of caries in panoramic radiographs, where lesions are often extremely small, low-contrast, and visually subtle compared with surrounding normal structures. These characteristics frequently lead to weak signals for the positive class. Coupled with the natural imbalance in lesion distribution, approximately 10% positive patches in the test set—the task becomes exceptionally difficult. Consequently, a moderate precision (≈0.31) and F1-score (≈0.46) are expected and acceptable, reflecting the trade-off between identifying all possible caries and minimizing false positives. The MCC values around 0.42–0.44 further confirm a good overall correlation between predictions and ground truth, even under imbalance. Importantly, the close agreement between cross-validation and test results demonstrates that the pipeline generalizes well and that the image-level splitting strategy effectively prevented data leakage. The strong recall and AUC show that the model captures clinically relevant patterns of caries despite dataset complexity. Overall, these findings present a benchmark for the newly introduced MD-OPG dataset, establishing a solid baseline performance for patch-based caries detection using a ResNet-18 architecture. Future research can build upon this benchmark to explore more advanced networks, multi-scale feature aggregation, or image-level decision fusion strategies aimed at improving precision without compromising sensitivity.

In previous studies, several approaches have been used to improve model focus in adult dentition, where teeth are more distinctly separated, such as contrast enhancement and cropping of individual teeth to isolate training samples [20], or utilizing a bitewing [21,22] and periapical radiographs [23] to better highlight relevant features.

**Figure 4:**
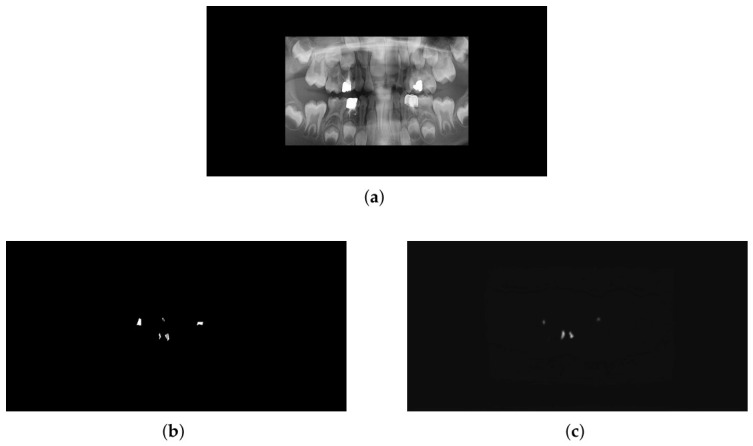
Segmentation results using the Attention U-Net model (Focal Loss). (**a**) Original input smile zone image. (**b**) Ground truth mask. (**c**) Predicted mask generated by the model.

In the next phase of this study, we evaluated the U-Net and attention U-Net model for the segmentation task using three different loss functions. As shown in Table 3, the U-Net model achieved its best results using the Focal Loss function. This performance aligns with the characteristics of Focal Loss, which effectively handles class imbalance through its adjustable parameters. In our dataset, where annotations are very small and negative class (non-caries) features significantly outnumber positive ones, Focal Loss excelled by mitigating the dominance of the majority class. The Dice loss function, based on the reported standard deviation and confidence interval, shows less stability compared to others. This confidence interval is quite significant even in the last 30 epochs, where the model is expected to have minimal inconsistency. In the experiment where we combined the Dice and Focal loss functions, we set their weights equally to ensure the same level of influence. The results show that this combination improves performance compared to the Dice loss alone, achieving relatively good stability in the last 30 epochs based on the confidence gap. However, the standard deviation throughout the 100 epochs is not ideal. With this in mind, the Focal loss function performed significantly better than the other two in the U-net architecture. Not only did it show greater stability in the last 30 epochs, but throughout the entire process, it demonstrated better consistency in terms of average Dice score, standard deviation, and confidence interval. According to Table 4 and the attention U-Net experiment, while the Dice loss achieved the highest Dice score, it exhibited lower stability compared to the other two losses. In this experiment, the focal loss demonstrated significantly better results again, not only in the last thirty epochs but throughout the entire training period. Although it did not achieve a Dice score higher than 91% during training, its standard deviation across all epochs was quite noticeable and significant, showing a very small gap within the confidence interval during the last 30 epochs. Additionally, in Figure 4c, we can see how effectively the mask generated by the Attention U-Net architecture using Focal loss highlights the desired regions. It successfully differentiates important and fine features from many complex characteristics. To evaluate the statistical significance of performance variations across different loss functions, the Wilcoxon signed-rank test was conducted on the Dice scores of 40 test images for each model. As summarized in Table 5, all pairwise comparisons within both the U-Net and Attention U-Net architectures yielded statistically significant differences (p<0.05). Specifically, for the U-Net model, the differences between all loss functions were highly significant Similarly (p<0.003). in the Attention U-Net architecture, significant improvements were observed when comparing Focal loss against both Dice and Dice+Focal losses, confirming that the choice of loss function significantly influences the segmentation performance for both network architectures.

Our segmentation results were found to be acceptable, even when compared to those that used a larger number of training samples and were conducted on adult panoramic radiographs [24]. In another study conducted by Xiaojie et al. [25], pediatric panoramic radiographs were utilized, and to prevent tooth overlap, each tooth was individually extracted so that the model could more precisely identify relevant features. Additionally, neighboring tooth features were fused for better classification. Despite this strong focus on individual teeth, our segmentation performance on the full panoramic image proved to be significantly more remarkable. In the method proposed by Asci et al. [26], segmentation was performed on pediatric OPG images from children aged 4 to 14, using a much larger dataset (around 1400 samples). However, their model did not achieve F1-scores higher than 81%.

### Limitation

There are some limitations that should be acknowledged. The size of the dataset is relatively limited; therefore, data augmentation strategies and robust architectures such as U-Net were employed to mitigate potential overfitting. Consequently, the reported results should be interpreted primarily as baseline performance estimates rather than statistically significant population-level conclusions. In addition, the MD-OPG dataset was collected from a single clinical site, which may introduce potential bias related to local patient demographics, imaging equipment calibration, and standardized clinical protocols. Future work should therefore include multi-center data collection to improve model generalizability and reduce possible dataset-specific biases. Another limitation is the absence of a direct comparison between model performance and dentists’ diagnostic performance. Although the dataset annotations were provided and validated by dental experts, this study did not include a reader study in which clinicians independently evaluated the same images for comparison with the automated predictions. Future research should incorporate reader studies involving dentists with different levels of experience to better assess the clinical usefulness of the proposed models. Finally, the imbalance in lesion distribution, particularly the predominance of proximal over occlusal caries, led us to formulate the classification task as a binary problem (normal vs. abnormal) rather than performing class-wise lesion classification and should be considered for future studies.

## 5. Conclusions

In this study, we propose a patch-based classification pipeline and two segmentation baselines (U-Net and Attention U-Net) specifically tailored for OPG images of children with mixed dentition. While the underlying architectures are standard, their application, training protocol, and evaluation on the MD-OPG dataset constitute a new methodological setup for pediatric caries detection in panoramic radiographs. The contributions of this study are two-fold: 1—Dataset contribution. We provide the MD-OPG dataset, a publicly available, expert-annotated OPG dataset of children with mixed dentition, including proximal and occlusal caries labels. This addresses the lack of benchmark data for this challenging developmental stage. 2—Methodological contribution. We design and evaluate a patch-based classification pipeline and segmentation baselines (U-Net, Attention U-Net) on MD-OPG. Although the architectures are standard, this is, to our knowledge, the first benchmark of these models on pediatric mixed-dentition panoramic radiographs, establishing reproducible baseline performance for future studies. The use of our introduced OPG dataset, carefully collected to protect patients’ confidential information, supports the development of AI-based and computer vision applications in dental health studies. The dataset’s unique characteristic of including images of patients with both primary and permanent teeth (mixed dentition) makes it a valuable resource for researchers and clinicians. Our study’s findings have significant implications for the field of dental health as they demonstrate the potential of deep learning-based methods in analyzing OPG images. The accuracy and efficiency of our approaches can aid in the early detection and diagnosis of dental anomalies, ultimately leading to improved treatment outcomes. Future studies can build upon our work by exploring the application of our methods to other dental imaging modalities and investigating the use of alternative strategies for patch extraction such as multi-scale sliding windows or fully end-to-end to improve the accuracy of our approaches. Additionally, future research directions building upon the MD-OPG dataset could explore hybrid strategies, integrating advanced signal processing and numerical modeling with deep learning approaches, as demonstrated in recent works [27,28,29], to further enhance the robustness, interpretability, and analytical capabilities for complex biomedical systems, ultimately leading to better health outcomes.

## Figures and Tables

**Table 1:** Descriptive Statistics and Metadata of the MD-OPG.

Field	Details
Data Type	Orthopantomogram (OPG) X-ray Images
Dentition Stage	Mixed Dentition
Subject Area	ML/DL-based pediatric dental disease analysis
Number of Samples	170
File Formats	PNG (images), JSON (annotations)
Age Group	3–12 years (mean age: 7.2)
Gender Distribution	50% Female/50% Male
Ethnic Group	Persian and Turkic
Annotations	P (Proximal), O (Occlusal), Z (Smile Zone)
Label Distribution	P: 716 (80%), O: 179 (20%)
Aspect Ratio	Full Image: 2:1, Smile Zone: 2:1
Date of Acquisition	2022–2024
Geographical Origin	Zanjan, Iran

**Table 2:** Performance of the proposed patch-level caries detection model on the MD-OPG dataset. Cross-validation results are reported as mean ± standard deviation, and the independent test set results are provided as single values.

Metric	5-Fold Cross-Validation	Independent Test Set
Accuracy	0.784±0.022	0.790
AUC	0.879±0.011	0.899
Precision	0.313±0.033	0.318
Recall (Sensitivity)	0.818±0.024	0.857
F1-score	0.452±0.033	0.464
MCC	0.413±0.024	0.436

**Table 3:** Results of the U-Net architecture on the test set with training on 3 different loss functions.

Loss Function	Metric	All Epochs (0–100)	Last 30 Epochs (71–100)
Dice Loss	Mean DS	0.6972±0.1249	0.7664±0.0566
	95% CI	(0.6724,0.7219)	(0.7453,0.7876)
	Best DS	0.8604	0.8604
	Last DS (Epoch 100)	0.8344	0.8344
Focal Loss	Mean DS	0.8812±0.0891	0.8991±0.0048
	95% CI	(0.8636,0.8989)	(0.8973,0.9009)
	Best DS	0.9093	0.9031
	Last DS (Epoch 100)	0.8923	0.8923
DiceFocal Loss	Mean DS	0.8515±0.0991	0.8808±0.0193
	95% CI	(0.8318,0.8711)	(0.8736,0.8880)
	Best DS	0.9071	0.9071
	Last DS (Epoch 100)	0.8921	0.8921

**Table 4:** Results of the Attention U-Net architecture on the test set with training on 3 different loss functions.

Loss Function	Metric	All Epochs (0–100)	Last 30 Epochs (71–100)
Dice Loss	Mean DS	0.8748±0.1327	0.9314±0.0071
	95% CI	(0.8485,0.9012)	(0.9287,0.9340)
	Best DS	0.9461	0.9461
	Last DS (Epoch 100)	0.9179	0.9179
Focal Loss	Mean DS	0.8970±0.0217	0.9076±0.0038
	95% CI	(0.8927,0.9013)	(0.9062,0.9090)
	Best DS	0.9147	0.9147
	Last DS (Epoch 100)	0.9067	0.9067
DiceFocal Loss	Mean DS	0.8839±0.1061	0.9215±0.0047
	95% CI	(0.8629,0.9050)	(0.9198,0.9232)
	Best DS	0.9343	0.9343
	Last DS (Epoch 100)	0.9208	0.9208

**Table 5:** Wilcoxon signed-rank test comparing segmentation performance across loss functions within each model.

Model	Comparison	*p*-Value
U-Net	Dice vs. Dice+Focal	p<0.001
	Dice vs. Focal	p<0.003
	Dice+Focal vs. Focal	p<0.001
Attention U-Net	Dice vs. Dice+Focal	p=0.007
	Dice vs. Focal	p<0.001
	Dice+Focal vs. Focal	p=0.007

## Data Availability

The dataset is available at https://doi.org/10.5281/zenodo.17786082. The code and additional figures are available at our GitHub repository (https://github.com/IASBS-AI-LAB/MD-OPG/tree/main (accessed on 14 April 2026)).

## Abbreviations

The following abbreviations are used in this manuscript:

MD-OPG	Mixed Dentition Orthopantomogram Dataset
OPG	Orthopantomogram
CNN	Convolutional Neural Network
